# A Recyclable Nanoparticle-Supported Rhodium Catalyst for Hydrogenation Reactions

**DOI:** 10.3390/molecules15053311

**Published:** 2010-05-05

**Authors:** Maria Michela Dell’Anna, Vito Gallo, Piero Mastrorilli, Giuseppe Romanazzi

**Affiliations:** Dipartimento di Ingegneria delle Acque e di Chimica del Politecnico di Bari, via E.Orabona, 4 I-70125 Bari, Italy; E-Mail: mm.dellanna@poliba.it (M.M.D.)

**Keywords:** hydrogenation, supported rhodium complex, nitroaromatics, halonitroanilines

## Abstract

Catalytic hydrogenation under mild conditions of olefins, unsaturated aldeydes and ketones, nitriles and nitroarenes was investigated, using a supported rhodium complex obtained by copolymerization of Rh(cod)(aaema) [cod: 1,5-cyclooctadiene, aaema^–^: deprotonated form of 2-(acetoacetoxy)ethyl methacrylate] with acrylamides. In particular, the hydrogenation reaction of halonitroarenes was carried out under 20 bar hydrogen pressure with ethanol as solvent at room temperature, in order to minimize hydro-dehalogenation. The yields in haloanilines ranged from 85% (bromoaniline) to 98% (chloroaniline).

## 1. Introduction

Heterogeneous transition metal catalysts leading to their efficient recyclability without a significant loss in activity as well as to facile separation of products from reaction mixtures without contamination of metal residues play an important role in economically and environmentally benign chemical processes. The immobilization of homogeneous metal coordination complexes onto an insoluble support is a very useful methodology for the synthesis of heterogeneous catalysts. Several supports have been employed for the immobilization of various homogeneous complexes including polymeric organic and inorganic supports [1,2,3,4,5,6,7].

Among heterogeneous catalysts, supported Rh complexes have been widely employed for promoting hydrogenation reactions [8,9,10,11]. In the field of the hydrogenation of unsaturated compounds the catalytic hydrogenation of aromatic halonitro compounds to yield anilines is very important, since haloanilines are a class of industrially interesting compounds used as starting materials or intermediates of many fine chemicals, such as dyes, drugs, herbicides, cosmetic products, pesticides and polymers [12,13]. The catalysts usually employed in the hydrogenation of halonitroarenes are based on transition metals [14], such as noble metals and Raney nickel, which is very sensitive toward the moisture in air and may burn.

Moreover, the hydrogenation of halo-substituted nitroaromatic compounds poses a serious problem due to the tendency towards hydro-dehalogenation, which is enhanced by amino substitution in the aromatic ring [15]. Various hydrogenation methods for nitroarenes with palladium [16,17,18], platinum [19,20,21,22,23,24], and ruthenium [25,26] heterogeneous catalysts have been recently proposed, all aimed at improving the selectivity towards the corresponding haloanilines and at minimizing the dehalogenation process.

Furthermore, the selective transfer hydrogenation of halonitroarenes promoted by transition metal catalysts in the presence of different hydrogen donors such as alcohols or formic acid and its salts has been reported [27,28]. Of remarkable interest is the novel transfer hydrogenation system developed by Yan and coworkers [29]. In this protocol, twelve “active hydrogens” can be transferred from water/ethanol system as the efficient hydrogen donor and used directly for the hydrogenation of halogenated nitrobenzene over Ru-Fe/C catalyst, obtaining *o*-chloroaniline with 98.0% selectivity at 99.8% conversion without dehalogenation.

**Scheme 1:**
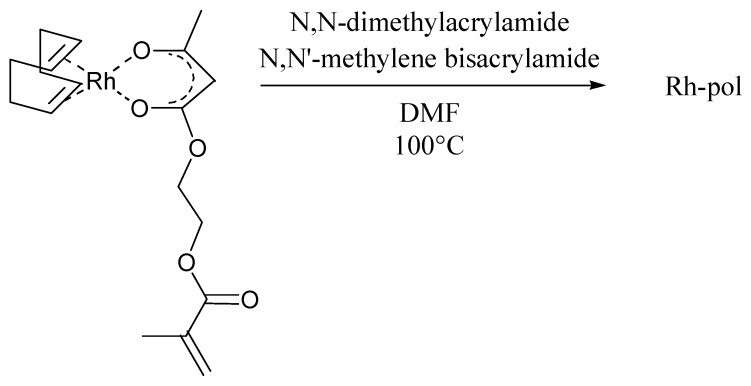
Synthesis of the supported rhodium catalyst.

In the framework of our research on hybrid catalysis, we have synthesised a polymerizable heteroleptic complex of Rh(I) bearing 1,5-cyclooctadiene (cod) and the anion of 2-(acetoacetoxy)ethyl methacrylate (aaema-) as ligands [30]. Rh(cod)(aaema) was copolymerized with *N*,*N*′-methylene bisacrilamide and *N*,*N*-dimethylacrilamide in dimethylformamide to yield a supported complex in which the catalytically active centres are dispersed onto an organic polymer matrix (Scheme 1).

This nano-structured catalyst has been also employed for the synthesis of poly(phenylacetylene)s with nanospherical morphology, reproducible and stable over time [31]. We report here on the catalytic activity of the above mentioned supported rhodium complex in heterogeneous hydrogenation reactions under mild conditions of several organic substrates such as olefins, unsaturated aldehydes and ketones, nitrobenzenes and nitriles. Moreover, we gained insight into the hydrogenation of halo-substituted nitroaromatic compounds at room temperature under 20 bar hydrogen pressure. 

## 2. Results and Discussion

The hydrogenation of cyclohexene at room temperature and pressure yielded cyclohexane in 2 h (entry 1, Table 1). The recyclability of the catalyst was verified by submitting the same recovered Rh supported catalyst to five subsequent cycles of this reaction and no appreciable loss in activity was observed. The results obtained using supported rhodium complex (Rh pol) in the hydrogenation of several organic substrates are summarized in Table 1.

In all cases good selectivities were achieved using mild reaction conditions and acceptable reaction times. It is worth noting that when the hydrogenation of (*R*)-(-)-carvone (entry 3) was carried out in methanol the selectivity for carvotanacetone was 77% after 6.5 h reaction. By performing the same reaction in CH_2_Cl_2_ (entry 4) the reaction rate decreased, but the selectivity improved.

**Scheme 2:**
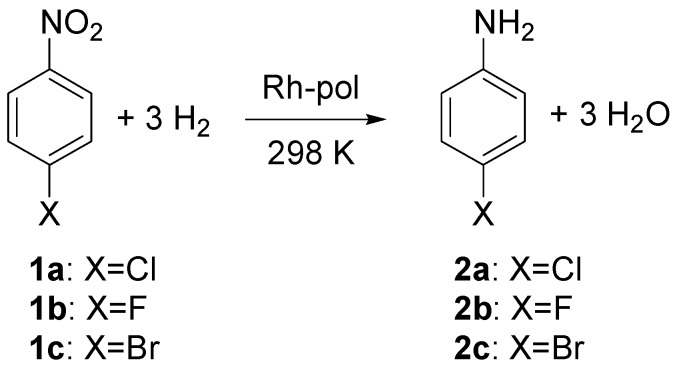
Hydrogenation of halonitroarenes promoted by Rh-pol.

The encouraging result obtained in the hydrogenation of nitrobenzene (entry 7) prompted us to study the hydrogenation of halonitroarenes promoted by Rh-pol. A paradigmatic substrate such as 1-chloro-4-nitrobenzene (**1a**) was submitted to hydrogenation at room temperature affording the corresponding *p*-chloroaniline (**2a**, Scheme 2), an important intermediate in the chemistry of dyes, drugs and pesticides.

No hydrogenation took place if the reaction was carried out at atmospheric pressure of H_2_, but high yields of **2a** (65-98%) were obtained raising the hydrogen pressure in the range 10-50 bar, as shown in Table 2.

It is apparent from Table 2 that the best yield in **2a** was obtained when the reaction was carried out under 20 bar H_2_ (98% **2a** after 72 h, entry 2). Under 50 bar H_2_, there was a significant decrease in the selectivity in **2a** (65%, entry 3) due to the reductive dechlorination of **1a** – in fact 35% of aniline was detected in the reaction mixture after 72 h reaction.

All the described reactions were carried out in ethanol, a typical solvent for the hydrogenation of unsaturated substrates. When the same reactions were carried out in methylene chloride, the formation of several hydrogenation by-products was detected regardless of the H_2_ pressure used. For example, under 20 bar H_2_ the yield in **2a** dramatically decreased down to 20% (at 100% conversion) after 72 h.

Subsequently, using Rh-pol as catalyst and the proper choice of process conditions, *p*-halonitrobenzenes **1a-c** were selectively hydrogenated to *p*-haloanilines **2a**-**c** (Scheme 2) giving high yields of the desired products (Table 3).

As summarized in Table 3, a long reaction time was required to convert **1b** into the corresponding fluoroaniline **2b** (95% in 7 days, entry 1). However, no dehalogenation product was observed. *p*-Chloronitrobenzene (**1a**, entry 2) was selectively hydrogenated into chloroaniline **2a**, as shown before. Only 2% of aniline, the product of reductive debromination, was found in the hydrogenation of bromonitrobenzene **1c**, affording, after 24 h, bromoaniline **1c** in 90% yield (entry 4).

The recyclability of the supported catalyst was tested by submitting the resin recovered from entries 2 and 4 to recycles. A higher hydrogenation rate was observed in both recycles (average t.o.f.’s pass from 2.2 h^-1^ to 6.3 h^-1^ in the case of **1a**, entries 2 and 3 and from 7.6 h^-1^ to 12.0 h^-1^ in the case of **1c**, entries 4 and 5), but the observed selectivity was slightly lower. In fact, at the end of the reaction, 6% (entry 3) or 8% (entry 5) of by-products other than aniline was detected by GCMS, including *p*-halo-nitrosobenzene and 4,4’-dihaloazobenzene.

The reason of the major activity of the resin in the recycle reactions may be the by-passing of the induction time necessary to the catalyst for its activation under 20 bar hydrogen pressure. Atomic absorption analyses of the recycled catalyst showed that the metal load in the resin was fully retained also after two reaction cycles.

## 3. Experimental

### 3.1. General

Rh(cod)(aaema) and its copolymer with *N*,*N*′-methylenebisacrylamide and *N*,*N*-dimethylacrylamide were synthesised according to literature methods [30]. The rhodium content of the resin was 5.59%. Atomic absorption analyses for the determination of the Rh content in the catalyst were performed with a Perkin Elmer 3110 instrument using a hollow cathode lamp. GLC analysis of the products was performed using a HP 6890 instrument equipped with a FID detector and a Supelcowax.-10 capillary column (30.0m x 0.25mm x 0.25 μm). GCMS data were acquired on a HP 6890 instrument using a HP 5MS 5% phenyl-methylsiloxane (30 m x 0.25 mm x 0.25 μm) coupled with a HP 5973 (EI, 70 eV) mass spectrometer. Conversions and yields were calculated by GLC analysis as moles of hydrogenated product per mole of starting nitro-compound by using dodecane as internal standard.

### 3.2. Hydrogenation reactions

A Schlenk tube charged with the unsaturated substrate (3.5 mmol) and the supported complex (containing 0.022 mmol of rhodium) in methanol or methylene chloride (3 mL) as reported in the caption of Table 1 was stirred under dihydrogen at 298 K monitoring the reaction course via GLC and GCMS. At the end of the reaction the heterogeneous catalyst was recovered by filtration, washed with methanol and diethyl ether, dried under vacuum and opportunely recycled. After duty the resin was analysed to determine the residual metal content. Negligible loss of rhodium was observed in all cases. In the case of reactions carried out at pressures other than ambient, a 50 mL steel autoclave equipped with an on-line spilling device was used. The hydrogenation of *p*-halonitrobenzenes was carried out in a 100 mL stainless steel autoclave equipped with a sampling device.

Typically, substrate (2.12 mmol), Rh-pol (substrate/Rh = 160 mol/mol) and dodecane (54 mg, as an internal standard for GLC) in ethanol (7 mL) were stirred vigorously under 20 bar hydrogen at room temperature and the reaction course was monitored via GLC and GCMS. At the end of the reaction, the supported catalyst was recovered by filtration, washed with ethanol and diethyl ether, dried under vacuum and opportunely recycled.

## 4. Conclusions

The nanoparticle supported Rh catalyst synthesised by copolymerization of Rh(cod)(aaema) with suitable co-monomers and cross-linkers showed a high activity and selectivity towards the hydrogenation reaction of several unsaturated substrates under mild conditions.

In particular, the hydrogenation reaction of *p*-halonitrobenzenes to *p*-haloanilines (halogen = F, Cl, Br) catalysed by Rh-pol under 20 bar hydrogen pressure at 298 K proceeded selectively without affecting the halo-groups on the aromatic ring. The recycled catalyst showed higher activity compared with the first cycle and did not leach out metal in solution.

## Figures and Tables

**Table 1:** Catalytic hydrogenations promoted by Rh-pol. Reaction conditions: substrate/supported rhodium = 160 mol/mol; T = 298 K.

Entry	Substrate	Time (h)	P_H_2__ (bar)	Conv. (%)	Products	Selectivity (%)
1 ^a^	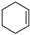	2	1	100	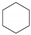	100
2 ^a^	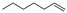	4	1	100	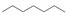	100
3 ^a^	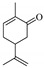 (−)-carvone	6.5	1	100	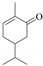 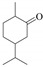	77 23
4 ^b^	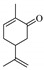 (−)-carvone	23	1	100	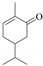 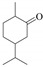	92 8
5 ^b^	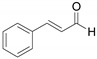	8	20	95	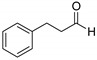 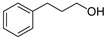	72 24
6 ^a^	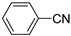	1	20	83	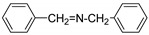	100
7 ^a,c^	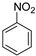	18	1	100	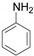	100

^a^: in CH_3_OH; ^b^: in CH_2_Cl_2_; ^c^: at 323 K.

**Table 2:** Effect of hydrogen pressure on the hydrogenation reaction of **1a** (2.12 mmol; solvent: EtOH, 7 mL; T = 298 K; substrate/Rh = 160 mol/mol).

Entry	Pressure (bar)	Time (h)	Conversion (%)	Selectivity for 2a (%)
1	10	72	65	100
2	20	72	100	98
3	50	72	100	65

**Table 3:** Hydrogenation of halonitrobenzenes under 20 bar of H_2_ at 298 K in the presence of Rh-pol (substrate: 2.12 mmol; solvent: EtOH 7 mL; substrate/Rh = 160 mol/mol).

Entry	Substrate	Time (h)	Conversion (%)	Product	Yield (%)
1	**1b**	7	100	**2b**	95
2 3 ^a^	**1a** **”**	3 2	100 100	**2a** **”**;	98 94
4 5 ^a^	**1c** **”**	1 0.5	98 100	**2c** **”**	90 90

^a^: Recycle of the previous run.
